# Dietary Epigallocatechin-3-Gallate (EGCG) Improves Nonspecific Immune Response of Chinese Rice Field Eel (*Monopterus albus*)

**DOI:** 10.1155/2023/6512136

**Published:** 2023-11-15

**Authors:** Haichao Deng, Huamei Yue, Rui Ruan, Huan Ye, Zhong Li, Chuangju Li

**Affiliations:** ^1^College of Fisheries and Life Sciences, Shanghai Ocean University, Shanghai 201306, China; ^2^Key Laboratory of Freshwater Biodiversity Conservation, Ministry of Agriculture and Rural Affairs of China, Yangtze River Fisheries Research Institute, Chinese Academy of Fishery Sciences, Wuhan 430223, China; ^3^Yangtze River Fisheries Research Institute, Chinese Academy of Fishery Sciences, Wuhan 430223, China

## Abstract

Epigallocatechin-3-gallate (EGCG) has been recognized as a potential additive for aquafeeds due to its beneficial biological functions. In order to evaluate the potential application of EGCG in Chinese rice field eel (*Monopterus albus*), six isonitrogenous and isolipidic diets containing 0, 25, 50, 100, 200, and 400 mg/kg EGCG were formulated and were fed to *Monopterus albus* (*M. albus*) for 9 weeks. The results showed that *M. albus* fed diets containing 0 and 100 mg/kg EGCG presented higher weight again and specific growth rate than the other groups. Fish fed with 25, 50, and 400 mg/kg EGCG displayed lower whole-body lipid content. Serum aspartate aminotransferase (AST) concentration significantly decreased in EGCG treated groups with the exception of 100 mg/kg group. Hepatic catalase (CAT) activity and glutathione (GSH) concentration decreased as EGCG level increased while malondialdehyde (MDA) concentration showed an opposite trend. EGCG supplementation resulted in a promoted lysozyme (LZM) activity and immunoglobulin M (IgM) level in the liver of *M. albus*. Furthermore, transcription of three immune related genes including major histocompatibility complex (*mhc-2α*), *hepcidin*, and interleukin-8 (*il-8*) mRNAs was upregulated by EGCG treatment; while transcription of interleukin-6 (*il-6*) and nuclear factor kappa-B (*nf-kb*) genes was downregulated. Results also showed a linear relation between EGCG inclusion level and parameters of AST, CAT, GSH, MDA, LZM, IgM, and immune-related genes transcriptions. In summary, it could be suggested that EGCG supplementation enhanced the nonspecific immune response of the Chinese rice field eel. Based on the broken-line regression analysis of IgM, the optimal dietary EGCG supplementation for *M. albus* was estimated to be 109.81 mg/kg.

## 1. Introduction

Herbal plants and their extracts have been extensively investigated for the aim of developing safe dietary additives. Those natural dietary additives improved growth performance, immune capacity, and antioxidant status in aquaculture species [1]. Additives of *Massa medicata*, *Crataegi fructus*, *Artemisia capillaries*, and *Cnidium officinale* have been reported to promote the growth, nonspecific immune response, lipid metabolism, and stress recovery of *Pagrus major* [2, 3]. In Nile tilapia (*Oreochromis niloticus*), nonspecific immune response and antimicrobial activity were enhanced by the addition of *Astragalus membranaceus* and *Lonicera japonica* [4]. Over 70 distinct plant extracts, such as curcumin, emodin, and chlorogenic acid, have been subjected to scrutiny in order to enhance the health and immune responses of Gilthead seabream (*Sparus aurata*) [5], Roho labeo (*Labeo rohita*) [6], and snakehead (*Channa argus*) [7].

Tea polyphenols (TPs), also known as catechins, are the major biologically active polyphenolic flavonoids extracted from tea [8]. Four kinds of catechins including (−)-epigallocatechin gallate (EGCG), (−)-epigallocatechin (EGC), (−)-epicatechin gallate (ECG), and (−)-epicatechin (EC) are mainly classified, among which EGCG is the most abundant accounting for 59% of the total catechins [9, 10]. These catechins have been reported to possess various physiological functions, such as antioxidative [11, 12], anti-inflammatory [13, 14], and antimicrobial properties [15, 16].

Considering the above diverse biological capacities, TPs are considered as promising feed additives in aquaculture species. In grass carp (*Ctenopharyngodon idella*), growth performance was enhanced by 500-mg/kg dietary TP supplementation [17]. Dietary TP addition promoted the anti-inflammation effect but attenuated nonspecific immune response in Koi carp (*Cryprinus carpio*) [18]. Dietary administration of EGCG at the inclusion level of 32 mg/kg revealed its potential role as an antioxidant and an immunostimulant in rainbow trout (*Oncorhynchus mykiss*) [19]. Growth performance, intestinal digestion, antioxidant status, and nonspecific immunity were all improved by dietary TP supplementation in juvenile hybrid sturgeon (*Acipenser baerii ♀ × A. schrenckii ♂*) [20].

Chinese rice field eel, *Monopterus albus*, is a protogynous hermaphroditic teleost species that is sex reversal from female, through an intersex stage, to male [21, 22]. This species is of considerable interest for commercial aquaculture in China, where its annual production reached 311,436 tons in 2021 [23]. Nevertheless, environmental stress and poor immunity have posed challenges to the rice field eel industry, resulting in the substantial economic losses. Therefore, the present study aimed to investigate the effects of dietary EGCG inclusion on growth performance, whole-body proximate composition, serum biochemistry, antioxidant activity, and nonspecific immune responses in Chinese rice field eel.

## 2. Materials and Methods

### 2.1. Experimental Diets

A basal diet was formulated using fish meal as the primary protein source, fish oil as the main lipid source, and *α*-starch as the primary carbohydrate source. Total, six isonitrogenous (430 g/kg, crude protein) and isolipidic (80 g/kg, crude lipid) diets were formulated to contain 0, 25, 50, 100, 200, and 400-mg/kg EGCG (designated as control, EGCG25, EGCG50, EGCG100, EGCG200, and EGCG400, respectively) (Table 1). EGCG, extracted from green tea, was provided by Mufan Biotechnology Co. (Xinyang, China), and the supplemental concentration for each group was determined based on a previous study [24]. All ingredients were mashed, passed through 80 mesh, and then mixed in a stepwise manner, with fish oil added lastly to ensure a homogenous mixing. The diets were stored at −20°C until used. EGCG content in the feed was determined by high performance liquid chromatography (HPLC) (Shimadzu LC-20A, Japan). In brief, 2 g of diet from each group were weighed and crushed, and then the diet was diluted to 10 mL with 70% methanol. The solution was extracted ultrasonically for 10 min. After a short centrifugation, 1 mL of supernatant was aspirated and an equal volume of stabilizing solution was added. The obtained solution was then filtered with a 0.45-*μ*m filter, and 10 *μ*L of the filtrate was injected into HPLC. A column (Capcell Pak C18 UG120, 4.6 × 250 mm, Shiseido) was used with a mixture of methanol and water (2 : 8 v/v, containing 0.1% phosphoric acid) at flow rate of 1.0 mL/min, and maintained at 35°C. Standard calibration curve was made to calculate the diet EGCG concentrations.

### 2.2. Feeding Management

Fingerlings of Chinese rice field eel with average weight of 19.74 ± 0.24 g and average body length of 28.28 ± 0.24 cm were purchased from a commercial hatchery (Qianjiang, China). The experiment was conducted indoors in two canvas pools (cubic: 4.0 m × 2.0 m × 1.0 m) at Liangzi Lake Experimental Site, Yangtze River Fisheries Research Institute, Chinese Academy of Fisheries Science. After 10 days of acclimation period, the fingerlings were randomly assigned to 18 net cages (cubic: 0.6 m × 0.7 m × 0.8 m), with 50 fish per replicate. The fish were hand-fed once per day (17:00–19:00) during the 9-week feeding trial. After 2 hr of feeding, any uneaten feed was collected, dried, and weighed. In the whole trial, the water temperature was maintained at 27.50 ± 0.12°C, while the dissolved oxygen concentration was kept above 5.00 mg/L and the ammonia nitrogen was maintained below 0.05 mg/L.

### 2.3. Sample Collection

After fasting for 24 hr, all the fish in each net cage were batch-weighed and counted to determine growth parameters including weight again (WG), specific growth rate (SGR), and feed conversion ratio (FCR). Additionally, the survival rate was calculated by counting the number of surviving fish in each net cage. Before sampling, fish were anesthetized with MS-222 (Sigma) and then nine fish per cage were dissected. Organosomatic indices including hepatosomatic index (HSI) and viscerosomatic index (VSI) were calculated by weighing the liver and viscera. Dissected livers of each treatment were divided into two parts, with one part for testing antioxidant capacity and the other part for testing the expression of immune-related genes, which were then dipped into liquid nitrogen and stored at −80°C until analysis. Furthermore, blood samples of three fish per net cage were collected by tail vein bleeding, which were then clotted at 4°C for 12 hr, centrifuged (3,000 *g*, 10 min) to obtain serum, and stored at −80°C for biochemical parameter analysis. In last, three fish per net cage were sampled and stored at −20°C to analyze the whole-body proximate composition.

### 2.4. Diets and Whole-Body Proximate Composition Analysis

Proximate composition analysis of both the test diets and whole-body samples were analyzed in triplicate following the standard method of the Association of Official Analytical Chemists (AOAC, 1995). In brief, samples were oven dried at 105°C for 22 hr to estimate moisture. The crude protein and lipid were measured by the Kjeldahl method and the Soxhlet method, respectively. Ash content was determined by burning the samples at 550°C for 6 hr in a muffle furnace.

### 2.5. Serum Biochemistry and Liver Antioxidant Capacity

Serum biochemical parameters including triacylglycerol (TG), total cholesterol (TC), alanine aminotransferase (ALT), and aspartate aminotransferase (AST) were detected using Mindray BS-460 analyzer (Mindray).

Hepatic antioxidant and immunity parameters including superoxide dismutase (SOD), catalase (CAT), glutathione (GSH), malondialdehyde (MDA), immunoglobulin M (IgM), and lysozyme (LZM) were determined using kits according to the manufacturer's instructions (Nanjing Jiangcheng Institute of Biological Engineering, China).

### 2.6. Quantitative Real-Time Reverse Transcription-PCR (qRT-PCR)

Total RNAs from three liver samples per treatment were extracted using Trizol reagent (Takara, Japan). Then the RNA was reverse transcribed with PrimeScript RT reagent Kit with gDNA Eraser (Takara, Japan) as outlined in the instructions. Real time PCR was performed in a volume of 20 *µ*L with SYBR green real-time PCR master mix (Takara, Japan) as instructed. According to the previous reports, ribosomal protein L-17 (*rpl-17*) was selected as the internal reference gene of rice field eel [25]. The gene-specific primers were summarized in Table 2.

### 2.7. Calculations and Statistical Analysis

The parameters were calculated as follows:

(1) $$\begin{array}{c} \text{Weight gain }\left(\text{WG},\%\right)=100\times \left(\text{final body weight }-\text{ initial body weight}\right)/\text{initial body weight}, \end{array}$$

(2) $$\begin{array}{c} \text{Specific growth rate }\left(\text{SGR},\%/\text{day}\right)=100\times \left(\text{Ln }\left(\text{final fish weight}\right)-\text{ Ln }\left(\text{initial fish weight}\right)\right)/\left(\text{the experimental duration}\right), \end{array}$$

(3) $$\begin{array}{c} \text{Feed conversion ratio }\left(\text{FCR}\right)=\text{feed consumed }\left(g\right)/\text{weight gain }\left(g\right), \end{array}$$

(4) $$\begin{array}{c} \text{Survival rate }\left(\text{SR},\%\right)=100\times \text{final number of fish}/\text{initial number of fish}, \end{array}$$

(5) $$\begin{array}{c} \text{Hepatosomatic index }\left(\text{HSI},\%\right)=100\times \text{liver weight}/\text{whole body weight}, \end{array}$$

(6) $$\begin{array}{c} \text{Viserosomatic index }\left(\text{VSI},\%\right)=100\times \text{visceral weight}/\text{whole body weight}. \end{array}$$

All results are presented as mean ± SEM (standard error of the mean). Data were analyzed using the software SPSS 25.0 (SPSS lnc.) with one-way analysis of variance (ANOVA), and Duncan's multiple comparisons were performed when there were significant differences (*P* < 0.05). For providing a comprehensive analysis, linear and quadratic regression analysis were applied to determine the relationship between EGCG concentration and measured parameters by SPSS 25.0 [26]. The broken-line model was applied to estimate the optimal dietary EGCG supplementation level by Origin 2021 software.

## 3. Results

### 3.1. Growth Performance and Morphometric Parameters

WG and SGR of *M. albus* fed control and EGCG100 diets were significantly higher than the other groups (*P* < 0.05) (Table 3). Fish fed EGCG25 diet showed the lowest WG and SGR values and the highest FCR value. VSI and HSI had a linear relation with the level of EGCG. Control group exhibited higher VSI than the EGCG supplemented groups (*P* < 0.05). HSI value was lower in fish fed EGCG400 diet than the other five groups (*P* < 0.05). The survival rate was not different among all the dietary groups.

### 3.2. Whole-Body Proximate Composition

Compared with the control group, whole-body lipid content decreased significantly in EGCG25 and EGCG50 groups, followed by a significant increase in EGCG100 and EGCG200 groups (*P* < 0.05) (Table 4). The ash contents of *M. albus* in control and EGCG25 groups were lower than EGCG100, EGCG200, and EGCG400 groups (*P* < 0.05). Whole-body protein and moisture contents were not remarkably changed by the dietary EGCG supplementation.

### 3.3. Serum Biochemistry

As shown in Table 5, serum TG concentration, ALT and AST activity had a linear relation with the level of EGCG. TG concentration gradually decreased with increasing EGCG supplementation level (*P* < 0.05). Fish fed EGCG50 and EGCG400 diets showed lower ALT activity than fish in the control group (*P* < 0.05). The activity of AST was the highest in fish fed control, while the lowest activity was recorded in EGCG400 group. Serum TC concentration was not affected by the dietary treatments.

### 3.4. Antioxidant Activity

**Figure 1:**
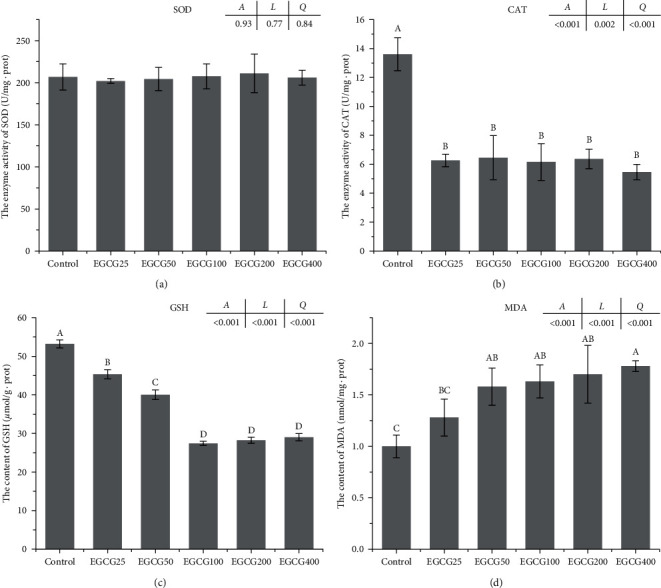
Activities of SOD and CAT, and concentrations of GSH and MDA in the liver of *Monopterus albus*. (a) SOD = superoxide dismutase; (b) CAT = catalase; (c) GSH = glutathione; and (d) MDA = malondialdehyde. Values are represented as means ± SEM (*n* = 6). Bars bearing the same or no letters are not significantly different. Means with different letters indicate significant differences in the treatments (*P* < 0.05). Letters *A*, *L*, and *Q* at the top of the plot show the *P*-value of ANOVA, linear relation, and quadratic relations, respectively.

EGCG supplementation led to decreased hepatic CAT activity compared to the control group (*P* < 0.05), however, no significant differences were observed in the five EGCG supplementation groups (Figure 1(b)). GSH showed a gradual decreasing trend as EGCG supplementation level increased and plateaued at over 100-mg/kg EGCG (Figure 1(c)). The lowest and highest MDA concentrations were observed in control and EGCG400 groups, respectively (Figure 1(d)). SOD activity was not influenced by EGCG supplementation (Figure 1(a)). There was a linear relationship between these parameters (CAT, GSH, and MDA) and the EGCG level in the diet.

### 3.5. Immunological Assays

**Figure 2:**
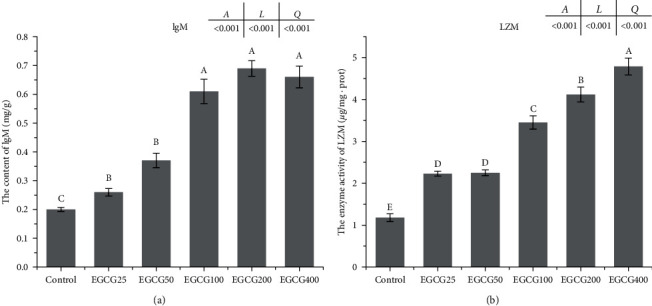
IgM concentration and lysozyme activity in *Monopterus albus*. (a) IgM = immunoglobulin M and (b) LZM = lysozyme. Values are presented as means ± SEM (*n* = 6). Bars bearing the same or no letters are not significantly different. Means with different letters indicate significant differences in the treatments (*P* < 0.05). Letters *A*, *L*, and *Q* at the top of the plot show the *P*-value of ANOVA, linear relation, and quadratic relations, respectively.

**Figure 3:**
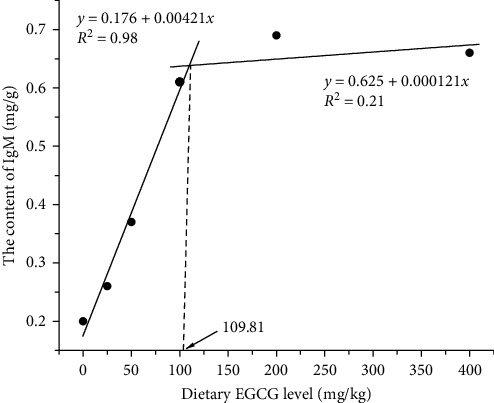
The broken-line regression analysis of IgM concentration in *M. albus* fed diets with different EGCG supplementation levels.

Compared to the control group, hepatic IgM concentration was increased as EGCG supplementation level increased (Figure 2(a)). LZM activity also showed an increasing trend by dietary inclusion of EGCG (Figure 2(b)). IgM concentration and LZM activity had a linear relation with the level of EGCG. Further, the broken-line regression analysis between IgM content and dietary EGCG level suggested that the optimal dietary EGCG supplementation for *M. albus* was 109.81 mg/kg (Figure 3).

### 3.6. Immune-Related Genes Expression

**Figure 4:**
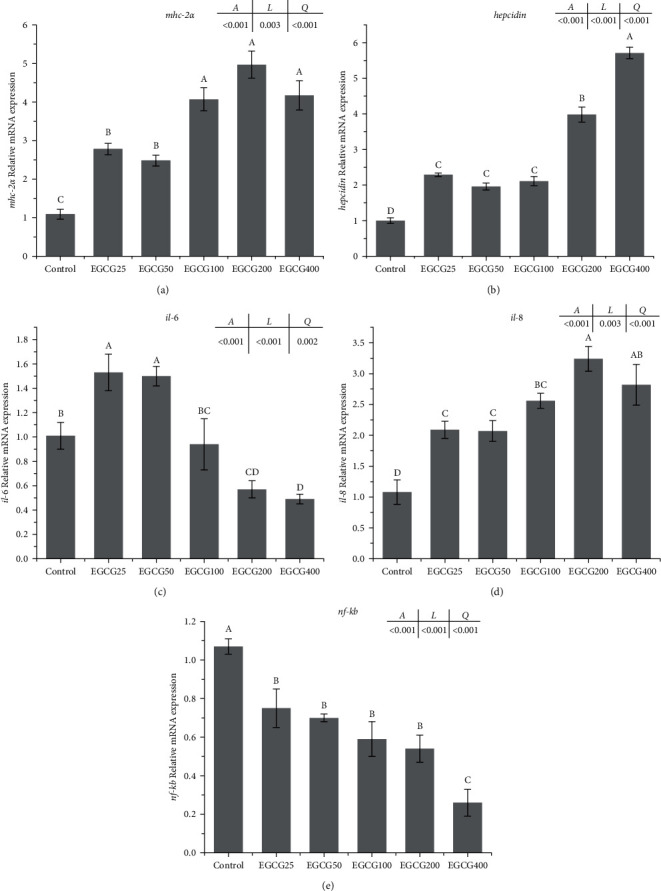
Expression of immune-related genes in the liver of *Monopterus albus*. (a) *mhc-2α* = major histocompatibility complex; (b) *hepcidin*; (c) *il-6* = interleukin-6; (d) *il-8* = interleukin-8; and (e) *nf-kb* = nuclear factor kappa-B. Values are presented as means ± SEM (*n* = 3). Bars bearing the same or no letters are not significantly different. Means with different letters indicate significant differences in the treatments (*P* < 0.05). Letters *A*, *L*, and *Q* at the top of the plot show the *P*-value of ANOVA, linear relation, and quadratic relations, respectively.

The lowest mRNA levels of major histocompatibility complex (*mhc-2α*) and *hepcidin* were observed in control group, and EGCG supplementation resulted in their upregulated transcription (Figures 4(a) and 4(b)). Fish fed EGCG400 possessed the highest *hepcidin* transcription in the liver followed by fish fed EGCG200 (Figure 4(b)). Compared to the control group, the interleukin-6 (*il-6*) mRNA levels were first upregulated by EGCG supplementation up to 50 mg/kg, but were subsequently downregulated at over 100-mg/kg EGCG (Figure 4(c)). Compared with the control group, dietary supplementation of EGCG caused the increase of interleukin-8 (*il-8*) transcription levels (*P* < 0.05), and the highest transcription was observed in fish fed EGCG200 (Figure 4(d)). The nuclear factor kappa-B (*nf-kb*) transcription in the liver of fish fed with EGCG were significantly lower than that in the control group (*P* < 0.05) (Figure 4(e)). There was a linear relationship between these parameters and the EGCG level in the diet.

## 4. Discussion

Dietary TPs supplementation has been extensively reported in aquaculture species. However, influence of dietary TPs on the growth performance of fish is still inconclusive. Growth performance was improved by dietary TPs in various fish species including black carp (*Mylopharyngodon piceus*) (50 mg/kg) [24], Nile tilapia (*O*. *niloticus*) (250 or 500 mg/kg) [27], grass carp (*Ctenopharyngodon idella*) (500 mg/kg) [17], and hybrid sturgeon (300 mg/kg) [20]. However, TPs did not significantly change growth performance in yellowtail (*Seriola quinqueradiata*) [28], rainbow trout [29], large yellow croaker (*Larimichthys crocea*) [30], and Koi carp [18]. In other species such as black rockfish (*Sebastes schlegeli*) [31], WG was reduced by the supplementation of green tea [31]. In this study, WG and SGR were decreased in dietary EGCG supplementation groups except in the EGCG100 group. This inconsistency might be due to the difference in fish species and nutrient status, as well as the concentration and composition of polyphenolic compounds [18]. Specific mechanisms underlying the effect of TPs on the growth of fish need further investigation.

In the current study, *M. albus* fed EGCG25, EGCG50, and EGCG400 diet showed lower body lipid contents than the other groups. The reduction of body lipid accumulation by EGCG was also observed in ayu (*Plecoglossus altivelis*) [32] and yellowtail [28]. It was reported that low concentration of EGCG could directly inhibit fatty acid synthase (FAD) and suppress FAD by upregulation of nuclear *β*-catenin [33]. Besides, high concentration of tea polyphenols could reduce the absorption of lipids in the gut, reducing the fat storage [34, 35]. Consistent with this, serum TG concentration decreased by dietary EGCG in Chinese rice field eel. TG is one of the main components in the lipid droplets [36]. This suggests that EGCG could decrease the level of blood lipid level and lipid accumulation, which was also reported in hybrid tilapia (*O*. *niloticus*) [35].

In the preceding investigation, dietary supplementation with EGCG increased the antioxidant activity in rainbow trout [17] and hybrid tilapia [35]. However, the present experiment exhibited a decline in the activity of CAT and GSH, and an elevation in MDA level in the EGCG treatments. It might indicate that EGCG was a pro-oxidant for *M. albus*, which increased the level of reactive oxygen species (ROS). In Korean bullhead (*Pseudobagrus fulvidraco*), dietary supplementation of 100-mg/kg EGCG reduced the enzymatic activities of SOD and CAT activated by copper [37]. EGCG autooxidizes and produces hydrogen peroxide in cell-culture media [38]. Of note, in this experiment, EGCG decreased, rather than increased, the ALT and AST activity in serum, implying that prooxidant actions of EGCG might be beneficial to *M. albus*. EGCG produced lower levels of ROS, stimulated multiple signal transduction pathways, and promoted cytoprotective mechanisms. For instance, the generation of ROS by EGCG led to the activation of AMPK, contributed to stimulation of lipolysis, apoptosis, reduction of endothelin-1 expression, and inhibition of gluconeogenesis [39–42].

In fish, lysozyme (LZM), antimicrobial peptides, and immunoglobulins are regarded as the components of innate and preventive immune productions and play crucial roles in the regulation of immune response [43]. LZM and IgM could eliminate bacteria and other invading pathogens and activate the complement system [44]. In the present study, LZM activity and IgM contents were increased continuously with the increase of dietary EGCG dose. It might indicate that nonspecific immunity capacity was enhanced by dietary EGCG in *M. albus*, which was in line with the previous studies showing increased lysozyme activity and complement content by dietary polyphenols supplementation in common carp (*Cyprinus carpio L*.) [45] and Asian sea bass (*Lates calcarifer*) [46].

On this basis, we further detected the transcription changes of immune-related genes. The major histocompatibility complex (MHC) plays an important role in binding and presenting processed peptide fragments [47]. Additionally, Hepcidin, a cysteine-rich antimicrobial peptide (AMP), has broad-spectrum antimicrobial properties [48, 49]. In the present study, it was found that EGCG upregulated hepatic mRNA transcriptions of *mhc-2α* and *hepcidin*. This result was consistent with that EGCG upregulated immune deficiency (IMD) pathway that regulated the expression of antibacterial-peptide gene expression and promoted innate immunity in Kuruma shrimp (*Marsupeneaus japonicus*) [50, 51]. The present study also showed that dietary supplementation with EGCG decreased the inflammatory transcripts of *nf-kb* and *il-6* in Chinese rice field eels. It was previously showed that EGCG could reduce the level of inflammatory factors such as IL-6 by inhibiting the activity of NF-*k*B [52–54]. Besides, EGCG inhibited inflammatory that usually accompanied by increasing oxidative stress [55, 56]. It should be noted that EGCG upregulated expression of *il-8*, which might be induced by ROS [57]. In summary, the effect of EGCG on inflammation was affected by many factors, and still needed further investigations.

In conclusion, the results of this trial suggested that dietary EGCG supplementation resulted in the increase of LZM activity and IgM concentration of the Chinese rice field eels. Transcriptions of immune-related genes such as *mhc-2α*, *hepcidin*, and *il-8* were enhanced, while the expressions of *il-6* and *nf-kb* were reduced by the dietary EGCG inclusion. Additionally, there was a linear relationship between these parameters and EGCG level. The optimal dietary EGCG supplementation for *M. albus* was 109.81 mg/kg by broken-line regression analysis of IgM.

## Figures and Tables

**Table 1:** Formulation and proximate composition of the experimental diets (g/kg, dry matter basis).

Feed composition (g/kg)	Control	EGCG25	EGCG50	EGCG100	EGCG200	EGCG400
Fish meal	450.00	450.00	450.00	450.00	450.00	450.00
Soybean meal	150.00	150.00	150.00	150.00	150.00	150.00
Wheat meal	80.00	80.00	80.00	80.00	80.00	80.00
*α*-Starch	160.00	160.00	160.00	160.00	160.00	160.00
Blood meal	20.00	20.00	20.00	20.00	20.00	20.00
Brewer yeast	50.00	50.00	50.00	50.00	50.00	50.00
Fish oil	40.00	40.00	40.00	40.00	40.00	40.00
Ca(H_2_PO_4_)_2_	15.00	15.00	15.00	15.00	15.00	15.00
Premix^a^	10.00	10.00	10.00	10.00	10.00	10.00
Choline	5.00	5.00	5.00	5.00	5.00	5.00
Earthworm meal	10.00	10.00	10.00	10.00	10.00	10.00
Mold inhibitor^b^	3.00	3.00	3.00	3.00	3.00	3.00
Sodium carboxymethyl cellulose	2.00	2.00	2.00	2.00	2.00	2.00
Microcrystalline cellulose	5.00	4.975	4.95	4.90	4.80	4.60
EGCG	0.00	0.025	0.050	0.10	0.20	0.40
Proximate composition						
Crude protein	429.80	429.40	429.80	433.50	430.30	432.40
Crude lipid	79.30	79.80	8.00	79.90	80.50	80.90
EGCG	0.00	0.029	0.052	0.075	0.17	0.35

*Note*. ^a^Vitamin and mineral premix composition (mg/kg): NaCl 50 mg, KI 1.5 mg, CoCl_2_ · 6H_2_O 2.5 mg, CuSO_4_ · 5H_2_O 15 mg, FeSO_4_·H_2_O 1,250 mg, ZnSO_4_·H_2_O 175 mg, MnSO_4_·H_2_O 80 mg, Na_2_SeO_3_·5H_2_O 1.0 mg, MgSO_4_·H_2_O 1,500 mg, vitamin B_1_ 12 mg, riboflavin 12 mg, vitamin B_6_ 8 mg, vitamin B_12_ 0.05 mg, vitamin K_3_ 8 mg, inositol 100 mg, pantothenic acid 40 mg, niacin acid 50 mg, folic acid 5 mg, biotin 0.8 mg, vitamin A 25 mg, vitamin D_3_ 35 mg, vitamin E 50 mg, and vitamin C 100 mg. ^b^Mold inhibitor: sodium benzyl alcohol 40%, gluconolactone 30%, sodium dehydroacetate 10%, sorbic acid methyl 10%, and disodium EDTA 10%.

**Table 2:** Primer sequences of RT-qPCR used in the experiment.

Genes	Forward (5′–3′)	Reverse (5′–3′)
*mhc-2α*	GTCCTCTTCTGTGCCCTCT	GAGCGTCCACTCCTCTGTT
*hepcidin*	GCCTTTATCTGCATTCTGG	CGCAGCCCTTGTAGTTCT
*il-6*	CTGTGACTACTGAGCGGAGAATGA	TGAGCTTCAAGGTCGGTTGC
*il-8*	TACTGGTTCTGCTTACTGTCGC	CAAATCTTTTGCCCATCCCT
*nf-kb*	GAAAAGCAATGACACCACTAAGACC	TTACCAATGAAATGCGAACACG
*rpl-17*	GTTGTAGCGACGGAAAGGGAC	GACTAAATCATGCAAGTCGAGGG

*Note*. *mhc-2 α =* major histocompatibility complex; *il-6 =* interleukin-6; *il-8* = interleukin-8; *nf-kb* = nuclear factor kappa-B; and *rpl-17* = ribosomal protein.

**Table 3:** Effect of EGCG supplementation on growth performance and morphometrical parameters of *Monopterus albus*.

Parameter	Dietary treatments						*P* value		
	Control	EGCG25	EGCG50	EGCG100	EGCG200	EGCG400	ANOVA	Linear	Quadratic
WG (%)	145.98 ± 1.79^a^	114.19 ± 2.39^c^	124.29 ± 2.37^b^	140.55 ± 4.53^a^	123.18 ± 1.96^bc^	123.94 ± 2.89^b^	<0.001	0.32	0.58
SGR (%/day)	1.73 ± 0.02^a^	1.46 ± 0.02^c^	1.55 ± 0.02^b^	1.69 ± 0.04^a^	1.54 ± 0.02^bc^	1.55 ± 0.03^b^	<0.001	0.34	0.61
Survival (%)	99.33 ± 0.67	94.67 ± 1.33	96.67 ± 1.76	98.67 ± 0.67	98.67 ± 0.67	100.00 ± 0.00	0.03	0.06	0.19
FCR (%)	1.71 ± 0.05^b^	2.02 ± 0.08^a^	1.99 ± 0.02^a^	1.74 ± 0.06^b^	1.87 ± 0.02^ab^	1.93 ± 0.06^b^	<0.001	0.56	0.82
VSI (%)	13.30 ± 0.34^a^	10.28 ± 0.12^b^	10.71 ± 0.32^b^	10.35 ± 0.13^b^	10.35 ± 0.30^b^	10.24 ± 0.25^b^	<0.001	0.002	<0.001
HSI (%)	5.34 ± 0.32^a^	4.50 ± 0.22^a^	4.25 ± 0.21^a^	4.43 ± 0.21^a^	4.36 ± 0.17^a^	4.08 ± 0.05^b^	<0.001	0.01	0.01

*Note*. Data are presented as means ± SEM (*n* = 3). WG = weight gain; SGR = specific growth rate; FCR = feed conversion ratio; VSI = viserosomatic index; and HIS = hepatosomatic index. Means in the same row sharing the same or no superscript letters are not significantly different. Means with different letters indicate significant differences in the treatments (*P* < 0.05).

**Table 4:** Effect of EGCG supplementation on whole-body composition of *Monopterus albus* (%).

Parameter	Dietary treatments						*P* value		
	Control	EGCG25	EGCG50	EGCG100	EGCG200	EGCG400	ANOVA	Linear	Quadratic
Crude protein	17.53 ± 0.21	17.35 ± 0.37	17.68 ± 0.24	17.82 ± 0.42	17.92 ± 0.17	18.07 ± 0.38	0.62	0.09	0.20
Crude lipid	6.29 ± 0.11^a^	5.77 ± 0.11^bc^	5.66 ± 0.10^c^	6.12 ± 0.10^ab^	6.21 ± 0.07^a^	5.70 ± 0.11^c^	0.002	0.35	0.32
Ash	2.14 ± 0.03^c^	2.20 ± 0.05^bc^	2.29 ± 0.00^ab^	2.35 ± 0.03^a^	2.37 ± 0.02^a^	2.40 ± 0.06^a^	0.002	≤0.001	<0.001
Moisture	71.67 ± 0.64	72.46 ± 0.63	71.84 ± 0.37	71.77 ± 0.41	71.84 ± 0.46	72.45 ± 0.60	0.28	0.20	0.15

*Note*. Data are presented as means ± SEM (*n* = 3). Means in the same row sharing the same or no superscript letters are not significantly different. Means with different letters indicate significant differences in the treatments (*P* < 0.05).

**Table 5:** Effect of EGCG supplementation on serum biochemical indices of *Monopterus albus*.

Parameter	Dietary treatments						*P* value		
	Control	EGCG25	EGCG50	EGCG100	EGCG200	EGCG400	ANOVA	Linear	Quadratic
TG (mmol/L)	0.68 ± 0.05^a^	0.47 ± 0.02^ab^	0.46 ± 0.02^ab^	0.40 ± 0.02^b^	0.36 ± 0.03^b^	0.36 ± 0.01^b^	<0.001	<0.001	<0.001
TC (mmol/L)	4.62 ± 0.08	4.12 ± 0.14	4.38 ± 0.28	4.37 ± 0.22	4.38 ± 0.25	4.18 ± 0.06	0.54	0.37	0.66
ALT (U/L)	3.43 ± 0.08^a^	2.9 ± 0.28^ab^	2.51 ± 0.13^b^	3.65 ± 0.44^ab^	2.86 ± 0.23^ab^	2.16 ± 0.15^b^	0.003	0.01	0.02
AST (U/L)	10.20 ± 0.41^a^	4.95 ± 0.30^d^	8.12 ± 0.52^bc^	9.06 ± 0.59^ab^	7.00 ± 0.55^c^	4.15 ± 0.21^d^	<0.001	<0.001	≤0.001

*Note*. Data are presented as means ± SEM (*n* = 6). TG = triacylglycerol; TC = total cholesterol; ALT = alanine aminotransferase; and AST = aspartate aminotransferase. Means in the same row sharing the same or no superscript letters are not significantly different. Means with different letters indicate significant differences in the treatments (*P* < 0.05).

## Data Availability

Data will be available from the corresponding authors by reasonable request.
